# Building sustainable talent pipelines: An ecosystem perspective on workforce integration and retention for internationally educated nurses

**DOI:** 10.1371/journal.pone.0356179

**Published:** 2026-08-20

**Authors:** Nuala Ryan, Elaine Berkery, Patryk Makowski

**Affiliations:** 1 Department of Management and Marketing, Kemmy Business School, University of Limerick, Limerick, Ireland; 2 Department of Work and Employment Studies, Kemmy Business School, University of Limerick, Limerick, Ireland; Ataturk University, Faculty of Pharmacy, TÜRKIYE

## Abstract

**Background:**

With the domestic supply of nurses failing to meet healthcare demands, sourcing nurses globally has become a key strategy in healthcare. Taking an Ecosystem approach, our study is designed to advance and deepen our understanding of how recipient country systems at the macro, exo, meso and microsystems layers can affect talent management practices for internationally educated nurses (IENs).

**Objective:**

The objective of this study is to answer the following research question: *How do the different layers of a host country’s ecosystem impact the talent management practices for internationally educated nurses?.*

**Methods:**

Using face-to-face semi-structured focus groups, data were collected from 21 IENs in Ireland. The results were managed and organised using the NVivo™ version 14 software, while Braun and Clarke’s reflexive thematic analysis approach was used to analyse the qualitative data based on their six-phase framework.

**Results:**

The experiences of IENs moving to Ireland suggest that at the macrosystem, national policy and structures around housing, early childhood education and visa deployment are adversely affecting their ability to work and stay. At the exosystem layer, they highlight clear recruitment ‘reality gaps’ and role mismatch on their arrival, as well as systems and language uncertainty. Finally, IENs describe how issues with organisational leadership and meso and microsystems can create a difficult day-to-day work environment.

**Conclusion:**

Through the ecosystem approach, we consider the broader macro and exosystems, as well as the more commonly researched micro and mesosystems, and their impact on talent management for IENs. The research findings show how national environments provide the context in which the exo, meso and microsystems operate and suggest the important need for policymakers as well as healthcare organisations to understand and adopt a holistic, multisystem view of how IENs are managed from a talent perspective.

## Introduction

Driven by a combination of global instability and climate change, ageing populations, and healthcare workforce shortages, the hiring of internationally educated nurses (IENs) is on the rise [1]. “ IENs are individuals who have completed a nursing education programme that is not located within the employer’s country [2]”. In their recent report on the international migration of doctors and nurses, the OECD suggests that the recruitment of internationally educated healthcare workers is often seen as a quicker solution to staff shortages than national training programs that can take years [3]. It is these national ecosystems that IENs are entering and are expected to grow, develop and remain, that domestically educated professionals are leaving behind due to structural issues and inadequate pay [4]. Research that provides evidence on the impact of the host’s ecosystem on integration, retention, and professional development for this cohort is critical to ensure IENs are not only recruited effectively but are also developed and empowered to reach their full potential.

Ecosystems are a network of multilayer, interacting parts or systems that are influenced by wider social and cultural processes. Bronfenbrenner and colleagues [5–8] describe human development as a constant interaction between the individual and their environment across four nested, interrelated dynamic layers: macro, exo, meso, and micro systems. The macrosystem is the outermost layer of influence acting on an individual and it includes the cultural, economic, societal and political context in which the other systems (exo, meso, micro) are embedded [5,7]. For example, policy and workforce regulation procedures around immigration in the USA have been found to influence community health workers’ effectiveness [9] and macrosystem decisions around public funding and research allocation can affect job security, staff shortages and limit educational or development opportunities in healthcare [10]. The exosystem is the layer of the individual’s environment that indirectly affects them, such as a person’s place of work, local government decisions or policies, or community organisations. The exosystem plays a vital role in the management of healthcare workers as decisions made by senior people in these systems can affect, for example, workload models that drive stress and burnout, which ultimately can impact patient outcomes [11]. Next, “a mesosystem comprises the interrelations among two or more settings in which the developing person actively participates” [7]. A strong mesosystem can provide stability, coherence and support across contexts, including work, family and community. Finally, the microsystem is the immediate environment in which an individual works and lives. It includes relationships and interactions in settings like the home, community and work. “Clinical microsystems are the small, functional, frontline units that provide most healthcare to most people” [12] and can include patient-provider relationships, care teams, family support units and organisational structures and settings.

An ecosystem approach can support a holistic understanding of how each layer of an organic system promotes talent management, including the development and retention of our healthcare workers. Recent studies have called on healthcare organisations to engage in talent management practices to help build more sustainable workforces [13]. Talent Management can be defined as “the systematic utilisation of human resource management activities to attract, identify, develop, and retain individuals who are considered to be talented” [14]. The concept includes HR processes and policies at the organisational level implemented to attract, identify, develop, engage and retain employees [15], highlighting a system that needs to retain, as well as recruit, to deal with any staff shortages. Within the healthcare system, talent management can support and strengthen the nursing cohort, preparing them for future leadership roles and vacancies as they arise [16]. As evidenced above, while the number of IENs registering in Ireland indicates that the Irish healthcare system is effective in attracting and identifying IENs, it appears to fall short on developing, engaging and retaining IENs. Talent management cannot be viewed in isolation, as it is inherently designed and implemented within an organisation, which itself functions within a wider societal and operating environment [17]. However, there is limited knowledge as to how wider society and operating environments influence talent management [18]. Research to date on talent management has tended to focus on the mesosystem, with limited research focusing on the macrosystem and microsystem [18]. To address this gap in the literature, this study aims to answer the following research question:

*RQ: How do the different layers of a host country’s ecosystem impact the talent management practices for internationally educated nurses?*

### Research context

The recruitment of IENs in Ireland mirrors that of the global context, where IENs are relied upon to fill national shortages. A report published by the OECD in 2023 highlights Ireland’s reliance on the recruitment of IENs, recording the highest number of IENs within the Irish nursing population at 51.8% [19], which is double the IEN population in Switzerland (25.8%, the country with the second highest number of IENs in 2023) [19]. Putting this into context, in 2023, 78% of new entrants on the nursing registrar in Ireland were IENs [20], with many IENs coming from India and the Philippines [21]. While little has been written on IEN’s experiences in the Irish healthcare sector, what has been written points to a situation where IENs do not intend to stay long-term, suggesting that the Irish system is failing to retain this strategically important category of workers. A previous study of IENs in Ireland reported that almost half (43%) of IENs were considering leaving Ireland [22]. Furthermore, research on IENs in Ireland identifies significant challenges, including inadequate pay, poor working conditions, feelings of isolation, limited access to further education and career progression, and the need to adjust to different cultural and professional practices [21–23]. In addition, research by Humphries et al [24] points out that only a few IENs had advanced into managerial grades, noting that perhaps this had stemmed from their reluctance to apply for such roles. The challenge therefore, lies in developing and retaining IENs in the Irish healthcare sector; a challenge that is significantly intensified by the absence of data regarding the experiences of these nurses after they enter the health service [25].

### Study setting

This cross-sectional study was conducted across a large hospital group in Ireland that serves a population of 380,000 people.

## Materials and methods

### Research design and philosophical approach

The aim of this study was to thematically review data gathered through focus groups relating to talent management practices for IENs in Irish hospitals. In order to uncover the subjective perceptions and experiences of IENs, qualitative methods were selected with face-to-face semi-structured focus groups as the data collection method [26]. We chose semi-structured focus groups because this format ensured we covered core topics with sufficient openness to let IENs interactively construct and share their experiences and meanings in a safe, exploratory setting, guided by the interpretive philosophical stance [26]. This approach allowed us to hear their lived experiences, e.g., how they made sense of the key topics discussed, rather than treating their views as fixed “responses” [27]. As reflexive TA researchers, we acknowledged that our professional backgrounds, values, and assumptions shaped every stage of this study, including question formulation, focus group facilitation, coding choices, and the writing process. None of us has personally migrated, but between us, we bring expertise in nursing research, qualitative methods, leadership, organisational behaviour, and talent management. Our expertise could predispose us to system-level or staff-level influences. Therefore, we recorded these assumptions in short reflexive memos, discussed them in peer debriefs, revisited the full transcripts to explore alternative interpretations, and documented any shifts in our understandings. Additionally, we had no employment or supervisory ties to participants or sites that were part of this research. Each of us had a different role within this project. NR was the lead researcher, co-conceptualised the study, and moderated all focus groups; EB co-conceptualised the project and helped establish its foundations; PM led the analytic process. All authors contributed to the iterative development of the themes and co-authored the write-up.

### Participants and recruitment

We used purposive sampling to collect detailed and rich data from IENs working and living in the mid-west of Ireland across the group’s hospital facilities. Purposeful sampling was specifically selected because our sample possessed valuable knowledge and experience crucial for our study’s objectives, while enhancing the rigour and trustworthiness of the data and results [28]. NR conducted four focus groups across the hospital in the group, with 21 IENs from countries including Poland, India, Croatia, Latvia, the Philippines, Zimbabwe, and Nigeria. The nurses were all at staff level, working full-time and were part of the hospital shift rotation programme. Participants were asked to get involved by senior members of nursing staff across the hospital group; however, these senior members of nursing staff were not present in the room or part of the data collection and analysis process. Focus groups were all homogeneous by staffing grade and role. To protect anonymity in this relatively small and tightly connected community of IENs, we deliberately did not collect personal demographic information (e.g., age or gender). Research has shown that internationally educated nurses experience personal vulnerabilities and challenges which can affect their integration and ultimately their retention [29]. Instead, we only noted the hospital site for each group and the details outlined above, ensuring that no individual could be identified from the data. The decision to restrict detailed disclosure aligns with ethical guidance on working with vulnerable populations, which emphasises the need for enhanced protections around consent, confidentiality and power imbalances [30].

### Procedures

We developed and employed a semi-structured focus group protocol with questions that focused on participants’ experiences with talent management practices based on their current roles within the organisations. Probing questions were asked during focus groups to deepen and clarify the discussion, if needed. Before each focus group, participants signed written consent forms and were informed of their right to withdraw at any stage during the focus group without penalty. Before starting, participants were given time to ask questions about the study’s aims, procedures, and to raise any confidentiality concerns they had. With the participants’ full knowledge and approval, each focus group was audio-recorded and professionally transcribed verbatim. Participants did not receive compensation for partaking in the study. Audio recordings were securely stored on password-protected files. Transcription of the data was carried out by a third-party provider listed among the university’s approved suppliers, ensuring compliance with the University’s data protection standards. As per Malterud [31], information power was considered in relation to our focused aim, specific sample, a sensitising theoretical lens, high-quality dialogue, and an interpretive reflexive thematic analysis strategy. After recruiting four groups (N = 21), additional recruitment was unlikely to deepen or diversify interpretive insights, so we closed data collection. All four semi-structured focus groups (one per hospital) were conducted by the lead researcher, NR. Each of these focus groups took place in private on-site meeting rooms and lasted approximately 60 minutes (mean duration: 56 minutes). Focus groups took place between June 8th and June 29th, 2023, during participants’ working hours at their workplaces, with only participants and the lead researcher present in the room. To report this study, we have followed a 32-item Consolidated Criteria for Reporting Qualitative Studies (COREQ) [32]. Full ethical approval was granted for this study by the hospital group (REC REF: 104/2021) and the home university (KBS Research Ethics Application: 2021_12_KBS_15).

### Data analysis

NVivo™ version 14 software was used to organise and manage the data. This software supported data organisation, retrieval, and visual thinking (e.g., cluster maps) as prompts for our reflection, while our analytic judgements remained researcher-led and grounded in close engagement with the transcripts, which is similar to the approach taken by Trainor and Bundon [33]. We followed Braun and Clarke’s [27] reflexive thematic analysis (RTA) approach to analyse the qualitative data using their six phases and Byrne’s worked example [34]. RTA is an iterative process in which researchers continuously move back and forth between phases to improve their understanding and interpretation of the data they work with. Braun and Clarke’s newest reflexive approach to TA emphasises the self-reflective element of why the data was interpreted in the way it was, which helped us be more transparent and understand the data and patterns better [34]. We adopted a hybrid reflexive thematic analysis, first coding inductively to construct themes as patterned meanings from the focus-group transcripts, and then organising the presentation of those themes deductively to align with our four-tier ecological framework (macrosystem, exosystem, mesosystem, and microsystem) [7,27].

**Fig 1:**
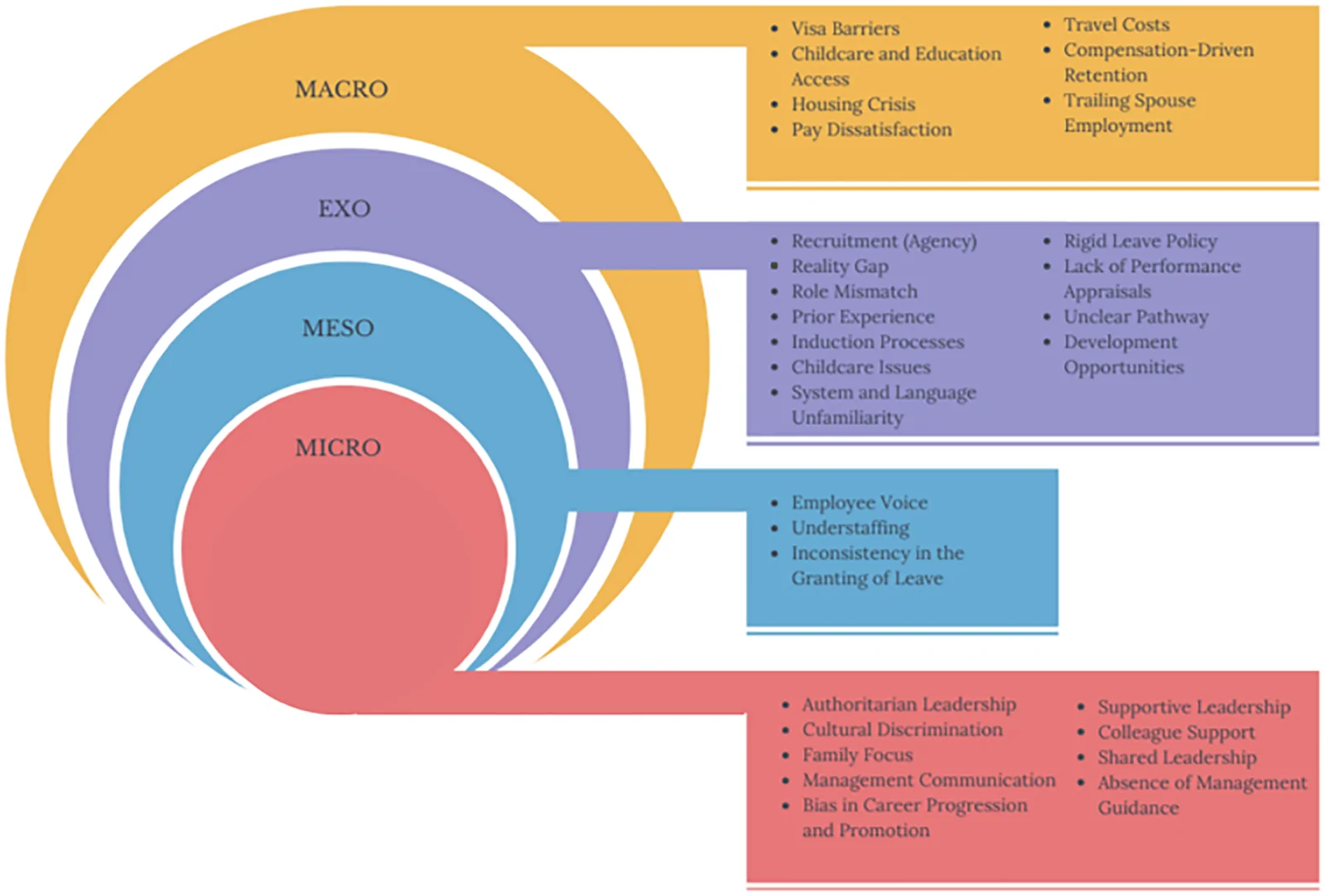
Adaptive Ecosystem model guided by Bronfenbrenner (1979, 1994, 1999; Bronfenbrenner & Morris, 2006).

The first phase of data analysis (familiarisation) involved active reading and re-reading of transcripts, writing reflective memos and notes, and sharing initial impressions with all authors to explore initial interpretations. During phase 2 (generating initial codes), PM coded every transcript using both semantic (surface) and latent (underlying) techniques while continuously revisiting and refining our code set across various stages. It involved tracking all merges, splits, and relabelling in an Excel™ code‑refinement sheet as we deepened our familiarity with the data. A total of 206 initial codes were generated in this phase. In the third phase (generating themes), PM clustered related codes into candidate themes and began sketching a thematic map. NVivo™ cluster maps were initially used as visual heuristics to stimulate reflexive questioning, rather than a means of guiding our analysis process, and explore alternative connections, where interpretive judgements were made through close readings of full data extracts, rather than relying on software algorithms. These reflections were discussed with NR and EB. During phase 4 (reviewing potential themes), we evaluated whether each candidate theme articulated a central organising concept and offered a distinct contribution to the overall narrative [34]. Thus, we held reflexive, collaborative review sessions, returning to NVivo™ extracts and original transcripts to split, merge, or discard themes based on coherence, and refining the thematic map. Phase 5 (defining and naming themes) was about discussion and debate among all authors, writing detailed definitions for each theme (central organising concept, boundaries, uniqueness, contribution) [27], refining theme names where necessary, and iteratively updating the final thematic framework (See Fig 1). Finally, phase 6 (writing the report) involved continuous movement between phases 5 and 6 to write an analytical narrative, incorporating illustrative, anonymised quotes into a cohesive report. Participants have been assigned numbers to maintain their anonymity when presenting the results.

### Quality and reflexivity

Historically, there has been much debate about quality in qualitative research. However, as Yardley [35] noted, it all comes down to the transparency and sensitivity of how the data has been collected, analysed, and reported. Yardley stresses the importance of not enforcing preconceived data categories, instead she outlines that researchers should evaluate the meanings behind participants’ words and expressions. To address this, we anchored quality to Yardley’s criteria of sensitivity to context, commitment and rigour, transparency and coherence, and impact and importance.

In practice, we (a) provided a description of the study setting and non-identifying, aggregate sample descriptors to support transferability while protecting anonymity of the participants, (b) we addressed analytic transparency through the utilisation of reflexive, analytic memos, decision logs, and evolving theme definitions and sustained engagement with the dataset to demonstrate commitment and rigour, (c) we described analytic decisions and provided illustrative extracts to support transparency and coherence, and (d) we articulated recommendations for policy, practice and theory to address impact and importance of this research.

**Fig 2:**
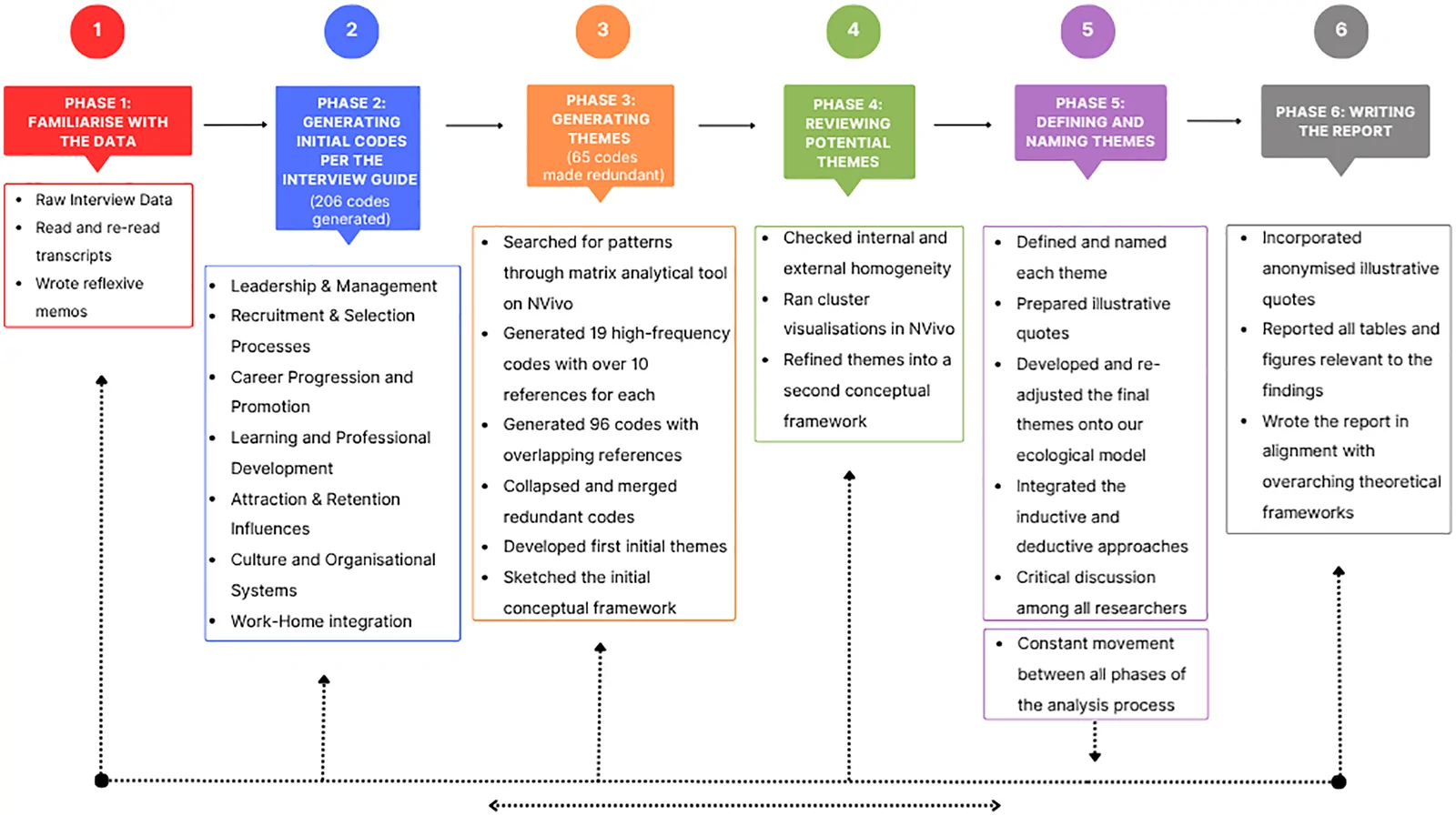
Reflexive thematic analysis workflow.

All focus groups were audio-recorded and sent to a certified third-party service for verbatim transcription. We then reread each transcript against the original recordings to improve transcript quality and support familiarisation. [27,34] Additionally, we developed iterative themes as a team, and held peer debriefs that helped us to identify assumptions, examine alternative interpretations, and prompted revisits to the full data extracts to support coherence and depth of reflexivity. For a visual illustration of the methodological process, see Fig 2.

## Results

**Fig 3:**
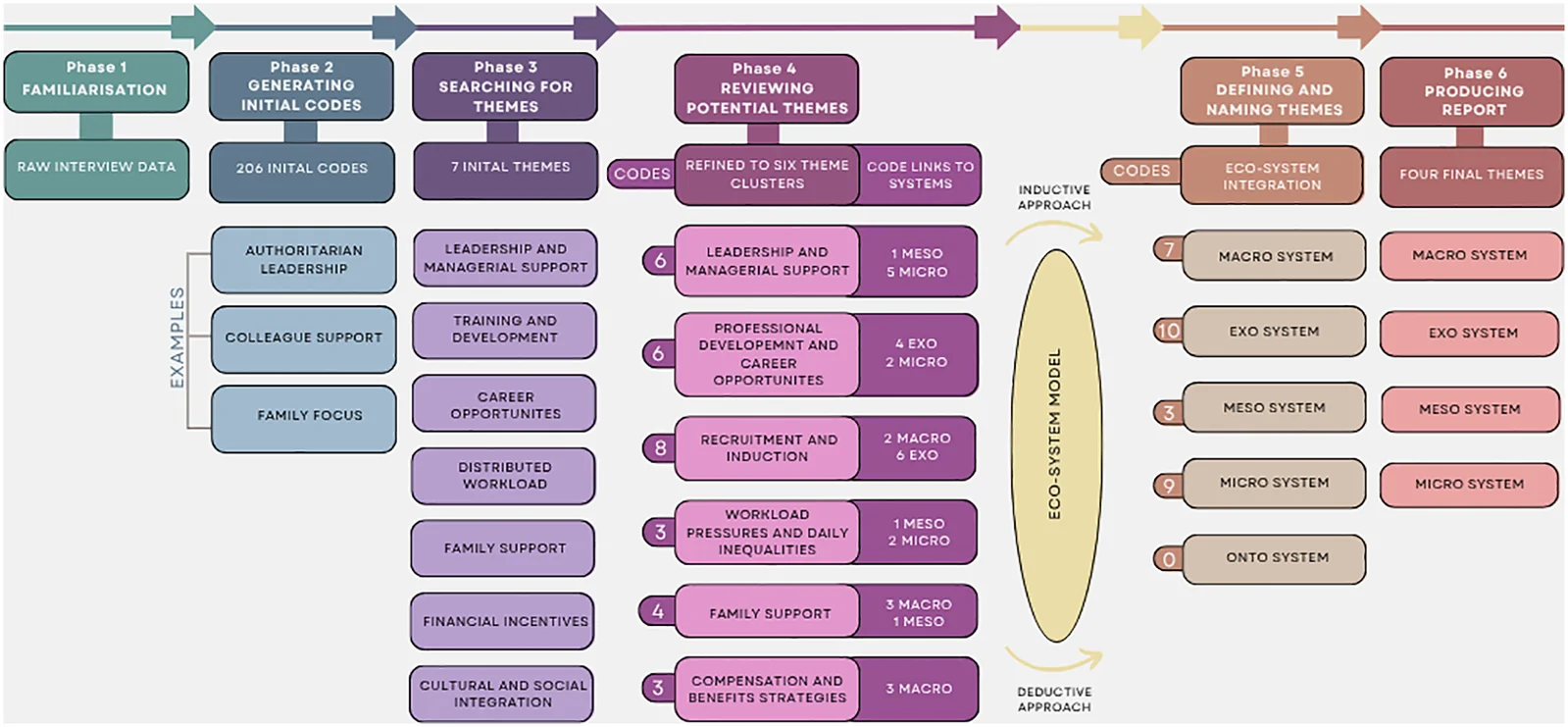
Final mind map of theme development.

In this section, we present the results of our analysis, considering each of the ecosystem layers, macro, exo, meso, and microsystems, individually to ensure a comprehensive exploration of the data. We reproduce participants’ words verbatim to retain their voice; however, where terms may be sensitive, we add brief analytic notes to contextualise their meaning. Under each of these headings, we discuss the themes constructed through our analytic process, shedding light on how distinct environmental layers influence workforce integration and retention for IENs. We also present our final mind map in Fig 3, which traces the development of our raw focus group data through all six analysis phases into the final ecosystem themes.

An overview of all themes and representative quotes is provided in Table 1.

**Table 1:** Classification of themes by ecosystem layer with representative quotes.

Ecosystem	Talent Management Themes	Sub-Themes	Quotes
**Macrosystem**	Attraction & Retention	Pay dissatisfaction	“Of course, the money will also make you leave.” “And no other allowances, no night allowances. No allowances, yeah. No Sunday pay, nothing.”
		Compensation-driven retention	“But to attract, the main thing I would say from India if somebody is coming, the main attraction will of course be money.” “I would say that’s the only reason that everyone is sticking around, money.”
	Work-Home Integration	Visa barriers	“After you get through your pain and you are well established in the health sector, you still have to wait for as long as six months or above before you could bring your spouse and kids.” “Finally I have my husband and three kids back home, and the six months was the toughest period of my life. At a point I had to tell my CNM, look, I am leaving this job because it was so impossible for my family to get their visas.”
	Work-Home Integration	Housing crisis	“So I think the most of them [IENs] are leaving here because of the accommodation.” “I know one of the friends are staying, three of them together in one -- three families together in one house. That’s no life, like. You need your privacy.”
		Childcare & Education access	“We have to depend upon the child minder all the time. Most of the families are migrating to Australia, the reason is that one.” “We had problems with school. That is a big issue. the older one we could not get a school anywhere. Nobody would have her. We were on the verge of going back”
		Trailing Spouse Employment	“One other thing which is, the spouses are not getting jobs here. That’s a big hassle.” “In Australia the jobs are better and easier to get. But here it’s very difficult, even though you are qualified, you don’t get the proper job here.”
		Travel costs	“We don’t have any families here. We don’t have any other support here, and we see all the family once in a year. We are spending an awful lot of money [to travel].”
**Exosystem**	Recruitment & Selection	Induction Processes	“Even if we have an adaptation of six weeks for a registration process, that six weeks adaptation won’t give us a guidance to understand what is happening in the system.” “The induction system should be demolished and it should be structured properly, professionally.” “My induction was two weeks and it was IV cannulation, venepuncture, everything, like. So then after two weeks you had all HC lines, everything was done and then we were like ready to go.”
		Recruitment (Agency) Reality Gap	“That’s how we are brainwashed because we know nothing about the country. We know nothing, you see. We don’t have any colleagues there, friends, like, who can tell us. I always tell my friends, no, come to the HSE (Health Service Executive) directly, it’s worth it.” “Unless and until you have the results in your hand, you are not getting a confirmation that you are going to be here.”
		Role mismatch	“Because I heard a few nurses telling like, you know, they want to go to [hospital name], they want to work in [hospital name] or [hospital name] where they have previous experience there, but they just ended in a different ward, wherever the short staffed are. So they are not happy in their workspace.” “They [management] said the system is totally different, you have to get into the nursing home and then after that you can move into the hospital.”
	Career Progression & Promotion	Unclear pathway	“I have an experience when I asked the CNM if I can go for this promotion and she said you have to be three years staying in Ireland, with this with experience. And I asked, where is it written? And she did not know that, but that was from her own words, which I think were not true.”
	Learning & Professional Development	System & language unfamiliarity	“One of the main barriers for international nurses would be the language. And we all learned in a different system. We are not familiar.,.”
		Lack of performance appraisals	“If you are a new nurse, working for six months, there is not even like one-on-one evaluation to say you are doing very well, you are adjusting or something like that, because it really boosts the confidence if you say, like, you fit very well in the system. Sometimes we do want to know.” “No, we don’t. It’s not happening nowadays.”
		Development opportunities	“They [management] encourage you to do further studies and they are always open for the higher position. I think people don’t go forward and take it. They are so good here.” “I was trying to apply for a course in UL. We need more of that.”
	Attraction & Retention	Prior experience	“If you come to Ireland your years of experience will be considered. That’s a huge benefit I considered before I come to Ireland. That’s the basic things that attracted me.”
	Support & Engagement	Childcare issues	“I think the HSE is recruiting machines because machines don’t need anything. If they are recruiting nurses, they should have think about, like, they’ll be having a family. They’ll need accommodation, therefore kids, they need an education.” “I just want to add, we come from a third-world country, but you know what, our hospitals have a creche. The creche would be literally on the hospital grounds, so you can see where your child is.”
		Rigid leave policy	“You are in Ireland, so you will be allowed two weeks’ annual leave per year, so you have to follow this.” “All the nights have to be covered before we are going [on holidays], and one of us, our colleague has to do all the nights, and when we come back we have to give them back several nights.” “For me, it’s the holiday is the biggest challenge.”
**Mesosystem**	Leadership & Management	Employee voice	“I would say that if there are representatives… from each, maybe each country ….. can voice some of the concerns occasionally with the management. Most of the problems from ten years ago are here, problems are still there.” “you are trying to do your best, support your colleagues and then you are bringing up problems and they are just ignored.”
	Attraction & Retention	Understaffing	“I never worked in the ward before I came over to Ireland. Here in the ward it is hectic. You will have to look after this many patients and there’s 10 patients who will be confused.”
	Support & Engagement	Inconsistency in the granting of leave	“It depends upon the managers.” “But I think lately they are not giving at all, they are not supporting us with holidays. It is a big pitfall.”
**Microsystem**	Leadership & Management	Authoritarian leadership	“They [management] kind of use the authority and they do what they want, not even considering how some of us feel. Like, they do what makes them comfortable.” “That’s their [management’s] decision, it’s a monarchical kind of thing. I don’t know where to go and ask why it was done.”
		Management communication	“…information dissemination about school, any like postgrad courses that will be available for international courses so that everyone is given out the information rather than individuals looking for that.” “When we come in as well, we don’t know what does a CNM1 do, what does a CNM2 do, what does a CNM3 do.”
		Shared leadership	“Even in my department, we share leadership, like, all are given equal chances for being a leader.” “Everyone is given all the responsibilities on the ward and everyone has to take little responsibilities. We all kind of share all responsibilities, and everyone is given an opportunity to develop these leadership skills.”
		Supportive leadership	“When I did, my CNM, you know, my CNM was really supportive to just promote the courses. It depends on the CNM too.” “I would say that, yes. If I need to compare a bit, yes, I would say here [in Ireland] it would be more democratic. That’s what I would feel.”
		Absence of management guidance	“And until something is happening, they [management] won’t do anything at all.” “And the lack of support from the management for me, for example, as well, in difficult situations you will be left by yourself.” “Sometimes it makes you feel you wish to go back.”
	Career Progression & Promotion	Bias in career progression and promotion	“It can be [bias]. And I have seen, I have attended three or four different interviews -- for the same position that I want to progress to, but the result I got, even after the preparation, the result I got wasn’t satisfactory for myself.” “I heard that someone I know who had less experience, or who hadn’t worked as much as I did, was selected. And that threw a huge disappointment to me and I feel that the next time I wouldn’t be bothered to attend.” “My colleagues in here who works with me, Irish colleagues …..told me, look, don’t get disappointment, they do have some candidates…., go for an interview to get an experience, and …. The next thing will come and try that.”
	Attraction & Retention	Family focus	“I am here so that maybe my family gets a good living. But that doesn’t necessarily mean that you don’t get discouraged, you know. You just keep going because you keep focusing on your goals.”
		Colleague support	“There was colleague support for me” “Some of them are very nice and they’ll tell us, like, you know, nurse ask me whether I need any support or something like that.”
		Cultural discrimination	“I promise you they will never do it to the fellow Irish nurses.” “If there is some other staff who works in the same place, in the same area, if only the colour difference is there, they won’t be pulled.” “Loads of favouritisms is what’s happening here.” “During the Covid time, ….. We asked them do we have the scrub uniform. Only the nurses won’t get any scrub, but there could be a cleaner, the kitchen staff, they are all wearing scrubs. We were told that there is a shortage of scrubs for nurses.”

### Macrosystem

The macrosystem is the broadest contextual layer that shapes talent management practices for IENs. Our reflexive analysis developed the overarching theme of attraction and retention with several sub-themes underpinning the talent management of this cohort.

The analysis foregrounded the interconnectedness of work and home domains, as well as less dominant themes such as visa barriers, the housing crisis in Ireland, limited access to necessary childcare and educational systems, challenges in securing employment for trailing spouses, and high travel costs affecting both individuals and their families. Government policies and procedures drive these challenges; participants cited significant barriers in visa administration and attainment, particularly regarding family reunification. One participant remarked*, “We have colleagues who have waited for more than a year to just have your family in. That’s a very big challenge”* (P2, FG2), while another described the process as *“the toughest period of my life”* (P1, FG2). Access to early childhood care and education was interpreted as another critical concern, with group members noting that nurses who have come to work in Ireland are increasingly migrating to Australia due to a lack of external childcare availability and limited family support. As one participant explained, Australia is favoured *“because they can bring the parents to look after the kids”* (P2, FG1) and in terms of impact they say *“we know that in the last two to three months nearly 20 families moved to Australia […] And they were here maybe 15, 16 years”* (P2, FG1). Additionally, the housing crisis has not only hindered the ability to find accommodation but has also led to cramped living spaces and the possibility of rent price gouging, as one person stated, *“There are people looting us. They are demanding more and more money”* (P4, FG3). Trailing spouse employment remains problematic as well, with one participant noting that *“my spouse was finding it difficult because they required Irish experience”* (P2, FG3), and exorbitant travel costs make it challenging to keep family connections strong, as illustrated by the comment, *“we see all the family once in a year. We are spending an awful lot of money [to travel]”* (P1, FG1).

The second major theme at the macrosystem level is centred on attraction and retention processes, specifically highlighting pay dissatisfaction and compensation-driven retention. Participants described how both the Irish tax system and the HSE’s pay structures contribute to financial dissatisfaction that may lead to migration from Ireland. One participant observed*, “I have colleagues who are planning to go to the US because the US tax system there is not like the Irish tax system. You do all the work and then all your money is gone”* (P2, FG2). On the other hand, some participants noted that competitive pay can be a significant attraction, citing for example, that nurses from India are often drawn to Ireland primarily for financial opportunities: *“I would say from India if somebody is coming, the main attraction will of course be money”* (P1, FG1).

### Exosystem

The exosystem relates to broader hospital and agency systems/processes that the nurse does not control day-to-day, but which shape their experience, e.g., staffing policies, training programmes. This theme captures the diverse challenges faced by IENs, which can be categorised under the following themes: recruitment and selection, career progression and promotion, learning and professional development, attraction and retention influences and work and home integration, each of which is sub-divided further.

Within the recruitment and selection theme, induction processes, recruitment (agency) reality gap and role mismatch were developed as sub-themes. Significant shortcomings were highlighted in the induction process, some participants reported delays in induction processes: *“I got it after maybe six or seven months after arriving”* (P3, FG4), while others felt that the induction process was too short “*You should give adequate time for everyone to adjust to the system. This is not a one week of time”* (P4, FG3), and because of the rapid induction process there was no gradual handover of responsibilities to new recruits, rather immediate parity with existing nurses was expected: *“Slowly you have to give the responsibilities... After one week you and the person who worked here for ten years both are equal”* (P4, FG3).

Furthermore, respondents highlighted gaps between promises made during the recruitment process: *“[…] they gave all this very nice green picture, […] but only thing is they gave us fake promises that we will be given accommodation […] But there was no connection between what they said and what was happening here”* (P1, FG3), and actual experiences upon arrival: “*[…] the job description is not well -- it’s not really spelled out, we do health care assistant jobs”* (P3, FG2). Additionally, there was disillusionment regarding the agency’s practices, including perceptions of financial discrepancies and a lack of transparency compared to expectations formed: *“My belief before I left my country that, oh, the whites they are more transparent and there is equity in the way they treat people. So, I could never imagine the agency would be paid such an amount and then they withdrew part of this and keep it in their pocket only to give you peanuts”* (P2, FG2).

Upon entering the system respondents felt confused by unfulfilled promises and the absence of clear support systems to help settle them into life in Ireland: *“Today I have seen so many of the people, they don’t know where to go”* (P4, FG3). Furthermore, upon arrival, many nurses ended up in roles or departments that did not match their clinical expertise or previous work experience, *“[...] I was in the nursing home because I had an experience gap of 1.5 year [...] Dementia was totally new to us. I hadn’t seen a patient with dementia in India”* (P2, FG4). Nurses with specialized or long-term prior experience often face frustration when assigned to unrelated work areas. *“So, when the agency told me about [hospital], they told me I am coming to an orthopaedic hospital. This is not an orthopaedic hospital...”* (P1, FG4).

Unclear pathways were highlighted by respondents under the theme career progression and promotions. There was widespread uncertainty about the criteria and process for professional advancement: *“I don’t know the process for that”* (P4, FG3). Respondents lacked clarity regarding how their previous experience at home translated into eligibility for senior roles in Ireland, with conflicting messages about the necessary tenure and qualifications required in Ireland for promotion to CNM posts: *“At home, we know the pattern, that after 10 years eligibility is built up for this post. But now I don’t know whether I can, whether I am eligible to apply for CNM post. I don’t know”* (P4, FG3).

Within the learning and professional development theme, system and language unfamiliarity, lack of performance appraisals and development opportunities were developed as sub-themes. Respondents struggled with unfamiliar language nuances and different healthcare systems, particularly when trying to adapt to new regulatory requirements. Respondents also felt the absence of regular performance appraisals hampered the ability to gauge and boost their confidence: *“If you are a new nurse, working for six months, there is not even like one-on-one evaluation to say you are doing very well, you are adjusting or something like that, because it really boosts the confidence…”* (P3, FG3). However, despite these challenges, there is institutional support for further education, with some respondents sharing positive experiences about further education and advancement opportunities: *“I did my master’s degree here […] funded by HSE […] I was encouraged to do it […] I also did a management course […] There are job opportunities as well, if you want to progress and go for a manager position”* (P2, FG4). However, the desire for more accessible and varied development courses persists: *“We need to have access to more smaller courses”* (P1, FG1).

Under the theme attraction and retention influences, a key incentive for coming to Ireland was the recognition and favourable calculation of years of experience, which is not always considered in other countries, *“Unlike UK, they don’t calculate our experience [...] they [HSE]* *calculate our experience, and we get a salary depending upon our experience [...] That’s a great attraction.”* (P3, FG4).

Finally, from a work-home integration perspective, family and leave challenges were discussed. Practical issues such as inadequate provision for childcare and rigid leave policies created significant obstacles for respondents, with some suggesting that recruitment should consider the full spectrum of a nurse’s personal needs, from family support to work-life balance: *“If they are recruiting nurses, they should have think about, like, they’ll be having a family. They’ll need accommodation, therefore kids, they need an education. This whole thing is as a series”* (P4, FG3). Some nurses had relocated from lower and middle-income countries (LMICs), where they had access to on-site childcare: *“I just want to add, we come from a third world country, but you know what, our hospitals have a creche… The creche would be literally on the hospital grounds...”* (P3, FG3). “Third world” is the term used by the participant. We interpret that it was the intention to compare the expectations of childcare provision, rather than to demoralise the participants’ home country.

### Mesosystem

The mesosystem in an ecosystem is the setting where talent management practices are designed and implemented. Feedback from the focus groups centred on broad themes such as attraction and retention processes, leadership and management practices, and the influence of organisational policy on work-home integration. Participants expressed deep concerns about understaffing, noting that a lack of sufficient staff directly compromised their ability to deliver quality care to vulnerable patients. One participant explained, “*You have a corridor of 18 patients and only two nurses to watch over them, administer medication, and care for very sick patients, it’s just too much”* (P3, FG2). Another emphasized the high nurse-to-patient ratio during night shifts, remarking, *“It could be at night-time when there are only three nurses, and if one is absent due to illness, only two nurses remain to care for 28 patients”* (P3, FG1).

Additional concerns focused on the unclear and inconsistent implementation of leave policies across the organisation. Participants pointed out that the management of leave varied significantly, with decisions often dependent on individual managers; one participant noted, *“It depends upon the managers”* (P3, FG2), while another observed that *“They will give three weeks of leave, but some wards will only allow two weeks”* (P2, FG1). When discussing leadership, many expressed frustrations that leaders and managers failed to listen to staff feedback or incorporate it meaningfully into organisational changes. One participant stated, *“Most of the problems from ten years ago are still here”* (P2, FG4), while another added, *“You bring up problems and they are just ignored”* (P3, FG4).

### Microsystem

The microsystem addresses how day-to-day managerial behaviours and interpersonal dynamics shape IENs’ experiences in the workplace. During the focus groups, participants shared their thoughts on leadership and management practices, especially how these directly affected their decision to stay or leave the organisation. In other words, participants described the decision-making process as highly centralised because of managers who exercised authority without consultation with the nurses. This top-down approach left nurses feeling powerless to question assignments or processes, deteriorating their professional confidence. The participants expressed that such a rigid hierarchy contributed to emotional exhaustion and a sense that one’s voice held little weight in shaping ward practices. One participant remarked: *“It’s almost like a modern slavery, I would say, God forgive me”* (P2, FG4). To clarify what the participant meant, we interpret this metaphor to express feelings of powerlessness and a strict hierarchy at work, rather than a literal statement about forced labour.

A recurrent concern was the physical unavailability of managers during critical moments. Rather than proactive oversight, IENs encountered managers only in response to crises, or not at all: *“You won’t see them all day. That’s their management style, I’d say”* (P1, FG1). Beyond presence or absence, IENs frequently expressed that when managers did engage, their understanding of frontline realities was limited. Participants suggested that this out-of-reality disconnection manifested in the uneven treatment and provided information about role definitions and professional development opportunities, which left some nurses uncertain about pathways for advancement or about the scope of senior clinical roles. One participant said: *“Not in touch with reality. No, they [management] are not. They are just in some different bubble”* (P4, FG4).

Conversely, when managers adopted a supportive stance, which was the case for some IENs, the participants described the influence on staff morale as positive. Particularly, nurses spoke appreciatively of those CNMs who solicited their ideas and actively promoted postgraduate courses for professional development: “*You are able to share your ideas […] But generally of course they [management] are very supportive”* (P2, FG2). The nurses also elaborated on the continuous distribution of responsibilities among all senior staff, which, in their view, builds competence and a sense of ownership and solidarity, which the participants valued highly: *“If ever I am working and I happen to be the most senior on that shift, then I have to take up the responsibilities of what is happening in the ward”* (P3, FG2).

However, the participants argued that they felt a pervasive sense of inequity in being promoted at their workplaces, noting that already obtained professional qualifications did not always translate into opportunities for advancement. IENs perceived that decisions were predetermined in favour of specific candidates (often those with local affiliations), damaging trust in the fairness of selection mechanisms and deteriorating their motivation to pursue further professional development: *“This is not the first time. This is again and again happening, so what’s the point in going when you are not going to get it”* (P2, FG1).

Finally, IENs frequently pictured setting up their careers in Ireland as a strategic choice to secure a better livelihood for their families back home. This forward‐looking orientation helped sustain them through periods of workplace stress or uncertainty by anchoring their efforts to broader life goals. Participants emphasised that what has also helped them sustain these challenging situations was colleague support who proactively offered assistance or answered questions, creating a safety net that mitigated feelings of isolation and built a collegial environment: *“If you call them [colleagues] for anything, or ask them about anything, they will stop and help and support”* (P1, FG4). On many occasions, participants explained how they observed subtle exclusionary behaviours from their managers, which ranged from dismissive attitudes to unequal access to resources. Such experiences, which contrasted sharply with the treatment of Irish staff, were seen as emotionally harmful and as signals that full belonging remained elusive: “*To be honest […] there is people showing faces because we come from a different country. I have seen that, and I have experienced myself”* (P3, FG4).

## Discussion

The aim of this study was to examine how the layers of a host country’s ecosystem shape talent management practices for IENs in Irish hospitals. Through the lens of Bronfenbrenner’s [7] ecological model, our findings suggest that forces ranging from broad national contexts to intimate interpersonal dynamics collectively shape talent management for IENs in Ireland.

At the macrosystem, our participants highlighted how national policies, healthcare labour market conditions, and societal attitudes set the stage for their experiences and directly influenced their morale. These perceptions are particularly intriguing since Ireland’s heavy reliance on IENs creates a context where internationally educated talent is indispensable yet potentially undervalued. Perhaps such a heavy reliance on IENs is why high-income nations often employ aggressive recruitment strategies to address staffing gaps, especially in highly needed professions in healthcare [36]. However, such quick fixes are not sustainable if systemic support is weak. Policy discourse in Ireland reflects the concerns of our participants, warning that active overseas recruitment will only succeed in the long term if matched by strong retention efforts [37]. In line with Bronfenbrenner’s notion of the macrosystem, our study highlights that government initiatives, ranging from visa pathways, childcare and education access to national integration programmes, critically influence IENs’ commitment to stay, a finding consistent with prior research [38]. At the exosystem level, our participants described how encountering bureaucratic hurdles (e.g., lengthy credentialing and registration processes), a rushed induction procedure, and unfulfilled recruitment promises indirectly influenced their work experiences. Participants in our study reported that these bureaucratic hurdles not only influenced their perception of their work but also contributed to increased stress during their transition into the Irish healthcare system from their native countries. These reports align with past research, which suggests that credential recognition and induction issues can hinder retention [39]. Bronfenbrenner’s exosystem represents external structures, such as regulatory bodies and community supports, which indirectly affect the individual and their families [40]. Likewise, our findings suggest that such external structures and supports have a substantial influence on nurse engagement and persistence. Conversely, IENs expressed that lacking these supports left them isolated and responsible for overcoming the many unknown obstacles on their own. Participants also described pervasive institutional barriers to career advancement where they were being passed over for promotions or speciality training due to institutional racism or unconscious bias in healthcare [41]. Our findings provide examples of how IENs were assigned less desirable duties or had their prior experience overlooked, each of which contributed to a decline in their morale. Conversely, many nurses shared positive experiences of allyship and organisational support, showing that improvement is possible. These insights underscore the importance of health institutions implementing fair policies, providing continuous support, and developing a culture within organisations that values IENs as valuable human assets rather than merely filling a gap by recruiting professionals who have just been introduced to the system and enabling IENs to thrive rather than merely survive in the host country [39].

At the mesosystem, our findings suggest that the day-to-day realities of hospital operations and policies influence the intention of IENs to stay. Specifically, our focus group participants identified regular understaffing issues, characterised by high nurse-to-patient ratios and minimal support staff, as a major driver of excessive workloads, stress, and compromised patient care quality due to poor workplace operational and employment regulations. Additionally, the participants argued that annual leave was granted inconsistently among staff by managers and was too unpredictable, which, on many occasions, prevented nurses from planning their personal time and maintaining a healthy work-life balance. The nurses described how these structural and unpredictable flaws were further worsened by a lack of formal feedback mechanisms not provided by management, resulting in a sense of voicelessness among staff who lacked their staff representatives to voice concerns and who felt that their concerns were often ignored by management. The findings from this study highlight that without inclusive human resource practices, fair staffing models, and a supportive work environment, IENs may become disengaged or choose to leave the organisation for good. The perspectives of our participants regarding these support structures align with previous research, which suggests that inclusive HR policies, equitable staffing, and positive work environments are essential for retaining IENs [41,42].

Finally, at the microsystem level, our findings suggest that colleague support and healthy relationships, close team dynamics, and personal coping resources play a foundational role in whether an IEN feels integrated or alienated on the job. Our findings resonate with Bronfenbrenner’s idea that the microsystem (one’s direct environment and relationships) is the most influential layer shaping an individual’s development [43]. In our study, IENs perceived positive microsystem interactions (e.g., friendships at work, mentoring relationships, supportive feedback) as contributing to a higher level of job satisfaction and intent to stay. Conversely, negative or exclusionary interactions at this layer were deeply corrosive. Several nurses in our study reported experiencing microaggressions and bullying from both patients and colleagues. These firsthand accounts reflect findings from other environments, where IENs face daily challenges at the microsystem, such as mistrust from patients and cliques among staff, which can lead to social and professional isolation [44]. What is worrying is that these experiences can accumulate over time and drive talented nurses away despite otherwise favourable working conditions. This fragile retention is also because participants emphasised how their personal and family situations are intertwined with their work environment. For instance, one nurse whose close family remained overseas spoke of feeling additional stress and loneliness after shifts, which led to overall negative feelings about the work experience. Conversely, those nurses with family or close friends in Ireland expressed that they found it easier to recharge and cope with workplace pressures. These perspectives only highlight the often-overlooked reality that the microsystem extends beyond the workplace and also includes staff’s personal life, which is an essential factor to consider for truly effective talent management practices [45].

Finally, our research indicates the importance of considering the cross-level interactions and dynamics of these systems in the talent management process. Sumner and colleagues [46] suggest that understanding the interconnectedness of the ecosystem layers is critical for developing effective talent management practices in organisations. Our analysis suggests dynamic pathways of interrelatedness between the layers of the ecosystem. For example, this quote ‘*“Finally I have my husband and three kids back home, and the six months was the toughest period of my life. At a point I had to tell my CNM, look, I am leaving this job, I have to go back home, strategize and then move to another country, because it was so impossible for my family to get their visas”* suggests outcomes of the sub-theme on visa barriers at the macrosystem level are strongly connected to human strain at the microsystem level. This can cause retention issues for the organisation at the meso level, ultimately disrupting the talent pipeline and limiting the organisation’s ability to attract, develop, and sustain skilled professionals over time.

### Recommendations for policy, practice and theory

This research holds significant implications for practice and policy as it relates to the management of IENs, while also contributing to the ecosystem and talent management theories. Firstly, at a policy level, recruiting IENs is both a drain on their country of origin [47] and an expensive process for their destination country [48]. As such, the onus must be on the recipient country to develop the appropriate support systems to maximise this transition. If a government’s continued strategy is to attract and employ IENs, to be successful, governments and policymakers need to reflect on decisions taken and their impact on the talent management for internationally educated workers. Considering, for example, the effect of the visa application process on these workers and perhaps enacting similar visa law reforms made in Australia for both the individual and family [49] could be beneficial. Healthcare organisations must advocate for policy challenges encountered by this cohort and call on governments to provide realistic policies that can support migration rather than hinder it. Practically, recognising that internationally educated workers face a different reality to domestic nurses is critical in the talent management process for this group of staff. Developing organisational structures and leadership practices that are based on an inclusive ideology is critical to maximise employee engagement and support the retention process for this group. Theoretically, evidence on the talent management of this cohort remains mainly at the mesosystem and microsystem layers of their ecosystem [18]. This research shows how a holistic, ecosystem approach to understanding the process of attracting, developing and retaining this cohort provides us with a much broader, more realistic understanding of how to manage and build a high-performing internationally educated workforce. Table 2 provides a sample action grid of implementable interventions for each of the different layers of the ecosystem based on the findings presented here.

**Table 2:** Action grid of implementable interventions for internationally educated nurses.

Ecosystem	Talent Management Theme	Action	Owner	Metric
Macrosystem	Attraction & Retention Attraction & Retention	Review the visa application process for IENs. This review should consider family reunification timelines, spouse workers’ rights and credential recognition pathways. Access to affordable childcare capacity and transitional housing schemes.	Healthcare providers and Government bodies to introduce law reform in this area- see the Australian reform as a best practice example. Recruitment agencies.	Family reunification within 6 weeks of nurses’ arrival. Create a dedicated access route to employment for spouses. Spouses should receive support within 6 weeks of arrival to help secure appropriate employment. IENs will have secured childcare and accommodation before the arrival of their family.
Exosystem	Attraction & Retention Attraction & Retention Learning & Professional Development Support & Engagement	Create a code of practice for recruitment agencies that standardises pre arrival job previews, standardised induction programmes for IENs upon arrival, clear and objective role matching to nurses’ qualifications and experiences. Standardised induction programmes that cover areas such as workplace orientation, job specific preparation, organisational structure, career pathways, developmental opportunities, safety and compliance, support and integration. Annual performance appraisals. Review leave policies for nurses.	Recruitment agencies. Healthcare provider Human Resource Departments. Line manager. Healthcare provider Human Resource Departments.	Standardised recruitment processes for all IENs regardless of the recruitment agency used. All nurses to partake in a standardised induction programme onsite within their first month of employment. Biannual performance appraisals in the nurse’s first year of employment, annual performance appraisals thereafter. Standardised leave policies for IENs.
Mesosystem	Leadership & Management Attraction & Retention	Nominated IEN advocates. Conduct a review of all staffing ratios.	Healthcare providers. Healthcare providers.	The appointment of an IEN advocate in each hospital site to create an employee voice for IENs. Fair and consistent staffing ratios across all wards.
Microsystem	Leadership & Management Attraction & Retention Attraction & Retention Leadership & Management Attraction & Retention	A review of communication systems. A review of Equality, Inclusion and Diversity (EDI) training programmes. The creation of a buddy/mentor scheme for IENs. A review of leadership presence standards across all shifts. Develop a micro learning course on country level documentation and safety protocols.	Healthcare providers. Healthcare providers. Individual wards. Healthcare providers. Healthcare providers.	Upgrade/creation of a dedicated portal containing all information nurses need on promotion guidelines, developmental opportunities available and funded training/college programmes. All staff to have undertaken dignity and respect training as well as the creation of a discrimination reporting mechanism in each healthcare setting. Each IEN to be assigned a buddy/mentor for their first year of employment. The visible presence of CMN2 or above on all shifts. All IENs to have completed this course within their first two months of employment.

## Conclusion

This study identified and examined the challenges faced by IENs upon entering the Irish healthcare sector within one hospital group in Ireland. Taking data from four individual focus groups, we applied an ecosystem framework, grounded in the talent management literature, to organise our findings. Unlike earlier research, which has primarily focused on individual and organisational level factors, neglecting the wider environment in which nurses are embedded, our approach offers a more holistic perspective, connecting the nurse, their immediate work setting, and the broader ecosystem.

Our findings show that despite significant investment by the Irish government to recruit IENs to fill the ever-growing vacancies in Irish hospitals, IENs face challenges not only on the job and within their workplace, but also in the wider ecosystem in which they are embedded. This highlights the critical need to consider the broader nursing ecosystem in which IENs are embedded to be considered in future discussions on the integration and retention of IENs. This critically important cohort of nurses for the Irish healthcare system is likely to return home or seek opportunities elsewhere.

This study emphasises the need for an overview of the current integration and retention policies of nurses in the Irish healthcare sector. On the strength of the findings we present in this paper, we call on healthcare leaders and policymakers to address the deficits in the nursing ecosystem. Only through meaningful reform and the culture of a responsive and inclusive working environment can the sector fully support the integration and retention of IENs.

These findings should be interpreted with the following limitations in mind. First, although data were collected from four hospital sites, they all belong to one single Irish hospital group; as a result, the cross-sectional nature of the data limits the ability to generalise the results across healthcare settings and in an international context. Future studies should incorporate data from multiple groups across different settings so that the findings are more generalizable. Second, while this study focuses on IENs, some of the issues identified may also affect Irish nurses working in the Irish healthcare system. Including both cohorts in future research would offer a more comprehensive ecosystem perspective on talent management in healthcare systems.

These findings may also be considered in terms of their broader applicability in relation to other healthcare systems that rely on IENs to address workforce shortages, underscoring the importance of considering the wider ecosystem in supporting their integration and retention. Similar nursing workforce and migration dynamics are evident in countries such as the United Kingdom, Canada, Australia, and New Zealand, all of which depend heavily on internationally educated nurses to sustain healthcare delivery. Consequently, the need for coordinated support across regulatory, organisational, professional, and social domains may have relevance beyond the Irish context, enhancing the transferability of these findings to other internationally reliant healthcare systems. Regardless of context, future sustainable workforce planning will require a shift from reactive international recruitment towards coordinated, long-term policy approaches that prioritise supportive infrastructures, inclusive workplace cultures, and clear career development pathways for IENs. Finally, given the reliance on IENs on a global scale, we recommend that future research on IENs consider the broader ecosystem when investigating their lived experiences in new destinations.
